# COMPARATIVE ANALYSIS OF PREOPERATIVE ULTRASONOGRAPHY REPORTS WITH INTRAOPERATIVE SURGICAL FINDINGS IN CHOLELITHIASIS

**DOI:** 10.1590/0102-6720201600010007

**Published:** 2016

**Authors:** Flávio KREIMER, Daniel José Dias CUNHA, Carolina Cavalcanti Gonçalves FERREIRA, Thais Menezes RODRIGUES, Lucas Gomes de Morais FULCO, Eduardo Sávio Nascimento GODOY

**Affiliations:** Integral Medicine Institute Prof. Fernando Figueira, Recife, PE, Brazil

**Keywords:** Laparoscopic, cholecystectomy, Cholelithiasis, Ultrasonography

## Abstract

**Background:**

Laparoscopic cholecystectomy is widely used for cholelithiasis. Abdominal ultrasonography often precedes this operation and can prove diagnosis, as well as helps in showing possible complications during the perioperative period.

**Aim:**

Evaluate the description of variables of gallbladder and bile ducts present in reports of preoperative abdominal ultrasonography in cholelithiasis comparing with surgical findings.

**Methods:**

Were studied 91 patients who underwent elective laparoscopic cholecystectomy with previous abdominal ultrasonography. Variables such as identification and amount of gallstones involved were evaluated, both in preoperative ultrasonography and during surgery to evaluate sensitivity, specificity, concordance and positive and negative predictive values.

**Results:**

The reports did not mention diameter of vesicular light (98.9%), organ distension (62.6%), gallstone sizes (58.2%), wall thickness (41.8%) and evaluation of the common bile duct (39.6%). Ultrasound had high values for sensitivity, consistency and positive predictive value for identifying the presence/absence of gallstones: 98.8%, 96.7% and 97.8% respectively. As for the amount of stones, ultrasonography showed agreement in 82.7%, negative predictive value in 89.1% and specificity in 87.7%, with lower values for sensitivity (68.2%) and positive predictive value (65.2%).

**Conclusions:**

The ultrasound reports were flawed in standardization. Significant percentage of them did not have variables that could predict perioperative complications and surgical conversion.

## Abstract (pt)

**Racional:**

Colecistectomia laparoscópica constitui tratamento padrão para colecistolitíase e ultrassonografia abdominal frequentemente a precede e pode prever possíveis complicações durante o período transoperatório.

**Objetivo:**

Avaliar a descrição das variáveis da vesícula biliar e vias biliares presentes nos laudos ultrassonográficos pré-operatórios na colelitíase, e compará-los aos achados cirúrgicos.

**Métodos:**

Foram incluídos 91 pacientes submetidos à colecistectomia laparoscópica eletiva com ultrassonografia abdominal prévia. Variáveis como identificação e quantidade de cálculos foram avaliadas, tanto na ultrassonografia pré-operatória quanto durante a operação para analisar a concordância entre eles.

**Resultados:**

Nos laudos não constavam diâmetro da luz vesicular (98.9%), distensão do órgão (62.6%), tamanho aproximado dos cálculos (58.2%), espessura da parede (41.8%) e avaliação do colédoco (39.6%). Ultrassonografia teve altos valores para sensibilidade, concordância e valor preditivo positivo para identificação da presença/ausência de cálculos; 98.8%, 96.7% e 97.8% respectivamente. Quanto à quantidade de cálculos, a ultrassonografia apresentou concordância, valor preditivo negativo e especificidade de 82.7%, 89.1% e 87.7%, respectivamente, com valores menores para sensibilidade (68.2%) e valor preditivo positivo (65.2%).

**Conclusões:**

Os laudos ultrassonográficos foram falhos no quesito padronização. Porcentagem significativa dos laudos não apresentava variáveis que pudessem prever intercorrências transoperatórias e conversão cirúrgica.

## INTRODUCTION

Cholelithiasis has worldwide scope, with estimated incidence of 1.39/100 person/year, varying little between populations. Predominates in females and in advanced age^16^^,^ 19. In the US, the third evaluation of the National Health and Nutrition Examination Survey estimated that 6.3 million men and 14.2 million women aged 20-74 years have cholelithiasis, of which 1-3% become symptomatic^9^. Its incidence is directly linked to ethnic factors, gender, age and genetics^25^. Biliary colic and acute cholecystitis are the main complications also occurring choledocholithiasis, perforation of the gallbladder (VB), pancreatitis, cholangitis and Mirizzi^2^ syndrome. Acute cholecystitis is suspected in patients with pain in the right upper quadrant or epigastrium, fever and leukocytosis; clear Murphy signal sustains the diagnosis^28^.

Laparoscopic cholecystectomy is the treatment of choice, with mortality and morbidity of approximately 0.5% and 10%, respectively^13^. This procedure has advantages when compared to laparotomy, such as reducing the length of hospital stay, incidence and intensity of pain in the postoperative period, better aesthetic effects and minor surgical trauma^14^.

To confirm the preoperative diagnosis, abdominal ultrasonography (USG) is the most frequently used exam, being diagnostic method with relative low cost, free of ionizing radiation, non-invasive and practical realization. This exam has estimated sensitivity and specificity of 84% and 99%, being gold-standart for the diagnosis of extrahepatic biliary diseases, detecting gallstones of 1.5-2 mm in diameter^10^^,^ 21 ^,^ 24.

Preoperative ultrasonography is valuable in determining surgical difficulties or even chance to laparotomic conversion^6^^,^ 12 ^,^ 15 ^,^ 18 ^,^ 20 ^,^ 24 ^,^ 27. The wall thickness and the diameter of the common bile duct may indicate greater difficulties in some steps of the operation^6^. The advent of surgical predictability brings benefits such as the recommendation for experienced surgical team and conversion to laparotomy^24^.

Observing that there is little information in the preoperative USG reports that could favor surgical approach and the existance of some divergent results comparing image and surgery, this study sought to evaluate the description of variables in the gallbladder and biliary tract that could suggest difficulties or the possibility of surgical conversion, shown in the reports, comparing sonographic and surgical findings.

## METHODS

This study was approved by the Research Ethics Committee in Human of Integral Medicine Institute, with the approval protocol number 4284-14. It is observational, prospective, descriptive, cross-sectional study performed in Oscar Coutinho Hospital, Comprehensive Medical Institute Professor Fernando Figueira, Recife, PE, Brazil. Data collection was conducted from August 2014 to May 2015. During this period, were evaluated 91 patients undergoing laparoscopic cholecystectomy on elective basis.

To perform this research were used: specific protocol, medical records, identification, age, weight, height, BMI, classification of general state according to ASA criteria, description of variables in gallbladder and biliary tract on ultrasound examination, comparison between the USG pre-operative and surgical findings, operative time and intraoperative complications.

The USG reports analyzed the sonographic parameters of the gallbladder and bile ducts possible to be measured by the method as follows: diameter of vesicular lumen (described numerically or by normal or abnormal expressions); VB distension; VB wall thickness (described numerically or by expressions thin or thickened); presence or absence of gallstones; description of the stones (single or multiple); approximate size of stones (described numerically or by expressions micro, small, medium, large gallstones); description of the location of the stones (fixed or mobile); description of the presence or absence of biliary mud; description of the presence or absence of perivesicular liquid; description of the conditions of the bile ducts; diameter of the common bile duct (described numerically or by normal or expanded expressions); VB topography; VB volume (described numerically or with normal or abnormal expressions) and overall VB dimensions (described numerically or with normal or altered expressions).

Intraoperative complications rates were described as: coliperitoneum, stones or stone fragments in the abdominal cavity, bleeding, gallbladder perforation, iatrogenic injury to the bile ducts, pus in the abdominal cavity and conversion to laparotomy.

Moreover, VB and biliary variables of sonographic examination were compared to the same surgical variables analyzed macroscopically by the surgical team as: VB wall thickness, conditions of the biliary tract, identification of stones and their number in VB (single or multiple). The data found in the operation were considered more reliable compared to ultrasound findings. The USG was evaluated for sensitivity, specificity, positive predictive value, negative predictive value and agreement to evaluate the variables analyzed separately.

Were included patients diagnosed with cholelithiasis, aged between 20 and 80 who would perform laparoscopic cholecystectomy, and previously submitted to ultrasonography of the upper or whole abdomen to confirm the diagnosis and evaluation before surgery. Were excluded the patients submitted to operation as urgency; with non-lithiasic disease; the ones that didn't have USG records; who had USG outside the state of Pernambuco and who were examined in more than one year from the operation date. The operations were performed by surgeons of the institution and USG by different professionals of the state of Pernambuco.

Statistical analyzes were performed using Stata version 12.1 (binomial test) and Microsoft Excel 2010 version.

## RESULTS

Preoperative characteristics were: women 87.9%, mean age 46.9 years, mean BMI 27.29 kg/m^2^ and ASA I in 56.0%, II in 41.75%, III in 2.25%.

The operations demanded average time of 70 min. In 51.6% of cases, intraoperative complications occurred. The predominant intraoperative event was the drilling of VB, which represented 35.2%. The complications rates are shown in Table 1.

**TABLE 1:** - Intraoperative complications

**Intraoperative complications**	**Incidence (%)**
Coliperitoneum	30.8
Stones/fragment in the abdominal cavity	19.8
Bleeding	15.4
Gallbladder perforation	35.2
Iatrogenic injury to the bile ducts	-
Pus in the abdominal cavity	03.3
Conversion	05.5

The average age in years and BMI (kg/m^2^) among patients in whom intraoperative complications occurred, was 49 years and 26.8 kg/m², respectively. Of these, 53.2% were classified as ASA I, 44.7% as ASA II and 2.1% as ASA III. By comparison, among the 48.4% who did not undergo intraoperative complications, the average age was 45 years and BMI of 27.7; 59.1% were ASA I, 38.6% ASA II and 2.3% ASA III.

Conversion to laparotomy occurred in five patients (5.5%). Of these, four were women and one man. The average age in years was 56 and the mean BMI was 24.8. Regarding the pre-operative anesthetic classification, 80% (n=4) were ASA II and one (20%) ASA I. The mean duration of surgery in these patients was 130 min.

Each participant performed prior USG, which report was reviewed by the surgical team before each procedure. Sonographic parameters (variables) of the gallbladder and biliary tract in preoperative USG reports are shown in Table 2.

**TABLE 2:** - Value rates of studied variables reported in USG

**Sonographic variables**	**Patients in whom it was measured (%)**
Lumen diameter of gallbladder	1.1
Gallbladder distension	37.4
Gallblader wall thickness	58.2
Identification of the presence/absence of gallstones	100
Number of stones (single/multiple)	95.6
Approximate stone sizes	41.8
Stones fixed/mobile	9.9
Biliary mud	3.3
Perivesicular liquid	3.3
Conditions of the bile ducts	73.6
Diameter of common bile duct	60.4
Gallbladder topography	34.1
Gallbladder volume	15.4
Overall dimensions of the gallbladder	7.7

Regarding the comparison in all 91 patients among the variables of the gallbladder and bile ducts obtained by ultrasound reports with the surgical variables measured macroscopically by the team of surgeons, it was shown that: 1) the thickness of the gallbladder wall was not measured in 41.8% of cases; 2) the conditions of the bile ducts (thin or dilated) were dismissed in 26.4% of reports; 3) the identification of the presence/absence of stones was the variable measured in all reports; 4) in 4.4% of cases the number of stones was not present. To compare these sonographic variables with the same features in the operation, the patients who did not have them in the reports were excluded.

Thus, to compare the variable identifying the presence/absence of stones, all 91 patients were included. If the amount of stones (single or multiple) two were excluded because they did not show them in operation and two others were also excluded because the USG did not have this variable. In the case of the wall thickness of the gallbladder, 38 reports did not have this parameter, so were excluded from the comparison. Regarding the conditions of the bile ducts, five patients were excluded because this parameter could not be displayed during the operation and 24 others were excluded by the absence of the variable in USG.

Comparing the sonographic and surgical findings, the operation was considered more reliable. The USG was evaluated for sensitivity, specificity, positive predictive, negative predictive and agreement values to evaluate each of these variables mentioned above separately. Furthermore, was calculated the confidence intervals at 95% for each value. These results are shown in Table 3.

Among the patients who underwent conversion to laparotomy, according to the macroscopic analysis of the surgical team, the wall of the VB of all these patients was thickened. However, according to the ultrasound examination of the gallbladder wall thickness, 40% (n=2) of the reports did not presented it. Another 40% (n=2) had thickened gallbladder wall in the report of the imaging study. Only one patient had a thin gallbladder wall according to the ultrasound examination (Table 4).

**TABLE 3:** - Agreement, sensitivity, positive predictive, negative predictive values and specificity of abdominal ultrasonography for specific variables

**Specific variables**	**Concordance**	**Sensibility**	PPV*	NPV**	**Specificity**
Presence or absence of stones in VB	96.7% (IC 95%: 90.7-99.3%)	98.9% (IC 95%: 93.9-100%)	97.8% (IC 95%: 92.2-99.7%)	-	-
Number of stones in VB (single or multiple	82.7% (IC 95%: 73.2-90%)	68.2% (IC 95%: 45.1-86.1%)	65.2% (IC 95%: 42.7-83.6%)	89.1% (IC 95%: 78.8-95.5%)	87.7% (IC 95%: 77.2-94.5%)
VB wall thickness of (thick or thin)	64.1% (IC 95%: 49.8-76.9%)	93.5% (IC 95%: 78.6-99.2%)	63% (IC 95%: 47.5-76.8%)	71.4% (IC 95%: 29-96.3%)	22.7% (IC 95%: 7.8-45.4%)
VB stones (single or multiple)	80.3% (IC 95%: 68.2-89.4%)	100% (IC 95%: 92.7-100%)	80.3% (IC 95%: 68.2-89.4%)	-	-

* PPV=positive predictive value; ** NPV=negative predictive value; VB=gallbladder

**TABLE 4:** - Patients with surgical conversion and its relation to the wall thickness by ultrasonography (n=5)

**Patients**	**Macroscopic thickness**	**Thickness by USG**
1	Thickened	Not observed
2	Thickened	Thickened
3	Thickened	Thickened
4	Thickened	Thin
5	Thickened	Not observed

## DISCUSSION

The surgical conversion rate (5.5%) is within the range shown in the literature (2.6% to 11.9%)^22^. Relationship between obesity or overweight and conversion was not observed, since these patients had mean BMI of 24.8 kg/m^2^; other studies show that it exists^11^^,^ 17 ^,^ 29 ^,^ 30. The average age among patients who were submitted to conversion was higher than the ones who did not undergo conversion, 56 and 46 years, respectively. Of the 11 men (12.1%) only one had surgical conversion. However, there is evidence that male gender is one of the predictive factors for conversion^8^.

The variable most described in USG reports was the presence of gallstones. This fact is probably due to the diagnostic suggestion of cholelithiasis, done by the surgeon^10^^,^ 21.

Conditions of the biliary tract (73.6%), diameter of the common bile duct (60.4%) and wall thickness of the gallbladder (58.2%) were the variables mentioned in the reports. The assessment of the common bile duct or common bile diameter is important because it is one of the factors that may indicate difficulties and surgical conversion^6^^,^ 18. The wall thickness of the gallbladder suggests also surgical difficulty and risk to conversion; wall thickness of 3-5 mm is important to be reported in USG^12^^,^ 15 ^,^ 18 ^,^ 20 ^,^ 24.

Although the parameters mentioned above should be part of USG, was noted that a significant percentage of the reports did not include them, as the wall thickness neglected in 41.8% of reports in this study. Also, the diameter of the gallblader lumen was not measured in 98.9% of cases; presence or absence of perivesicular liquid in 96.7%; distention of the organ in 62.6%; approximate size of stones in 58.2%.

So, some factors may suggest the possibility of conversion to laparotomy and it can be evidenced by ultrasonography. The indicative of difficulties are: thick gallbladder wall, distension of the gallbladder, size of gallstones, diameter of the common bile duct, impacted stones in biliary tract, perivesicular liquid and diameter of VB^6^^,^ 12 ^,^ 15 ^,^ 18 ^,^ 20 ^,^ 24 ^,^ 27. Although there is no consensus in the literature about the importance of all parameters together mentioned above, different studies agree on the role of preoperative ultrasonography to predict difficulty and/or surgical conversion. Some authors consider even that radiologists should indicate the risk to conversion in the USG report^4^^,^ 27.

In order to improve the results of laparoscopic cholecystectomy were formulated preoperative scores to indicate difficulty degrees for the surgical procedure. In addition to the ultrasound factors, there are clinical parameters that can predict difficulties. They are: male gender, age over 70 years, biliary colic for the last three weeks before surgery, acute cholecystitis history treated conservatively, previous surgery of the upper abdomen, pain and stiffness in the upper right quadrant. These scores have been proposed for clinical and sonographic concurrently, and can help in surgical planning^7^^,^ 13 ^,^ 18 ^,^ 23 ^,^ 27 ^,^ 29.

Regarding the comparison between the variables of the gallbladder and biliary tract of sonographic reports with the same surgical variables measured macroscopically by the team of surgeons, it was found that USG has high values ​​for sensitivity, consistency and positive predictive value for the presence/ identification/absence of stones, 98.8%, 96.7% and 97.8% respectively. In the literature, the sensitivity value for stones identification ranged from 85-98%^1^^,^ 5 ^,^ 26. Regarding the number of gallstones (single or multiple) the USG has agreed in 82.7%, with negative predictive value in 89.1% and specificity in 87.7%. In relation to sensitivity and positive predictive value for the same parameter, the present study found 65.2% and 68.2%, respectively. In this sample, the ultrasound examination was better for the identification of stones than for its number, considering the confidence interval. In the literature review was found only one study that mentioned USG sensitivity on the stone number; it was 74%^3^.

For the evaluation of the gallbladder wall thickness, was observed in this sample considerable sensitivity (93.5%), but low specificity (22.7%) of the ultrasound examination. The assessment of bile ducts condition USG showed sensitivity of 100%, positive predictive value and agreement of 80.3%. The sample obtained for comparison of the last two variables was small, given the large amount of USG without reporting them. In addition, the macroscopic evaluation does not have the reliability and accuracy of pathological analysis to thickness of the gallbladder wall (thin / thickened) and conditions of the bile ducts (thin/dilated). Pathological analysis was not performed in this study. In the literature review, there are no studies analyzing sensitivity of USG for thickness of the gallbladder wall and either to the conditions of the bile ducts.

## CONCLUSION

USG reports are flawed in standardization, and often despise variables that have importance to surgeon, signalizing surgical difficulties and conversion. Abdominal ultrasonography showed high surgical concordance for identification of gallstones, but not to their number. The wall thickness of the gallbladder, when mentioned in USG, is important factor to predict operative difficulties
